# Abatacept downregulates Fcγ receptor I on circulating monocytes: a potential therapeutic mechanism in patients with rheumatoid arthritis

**DOI:** 10.1186/s13075-022-02886-8

**Published:** 2022-08-13

**Authors:** Ryosuke Fukue, Yuka Okazaki, Takahisa Gono, Masataka Kuwana

**Affiliations:** Department of Allergy and Rheumatology, Nippon Medical School Graduate School of Medicine, 1-1-5 Sendagi, Bunkyo-ku, Tokyo, 113-8603 Japan

**Keywords:** Rheumatoid arthritis, Abatacept, Monocytes, Fcγ receptors, Anti-citrullinated peptide antibody, Cytokine

## Abstract

**Background:**

Abatacept is a recombinant fusion protein composed of the extracellular domain of cytotoxic T-lymphocyte antigen 4 and the Fc portion of immunoglobulin (Ig) G. The mechanism of action of abatacept in rheumatoid arthritis (RA) is believed to be competitive inhibition of T cell costimulation mediated by the binding of CD28 to CD80/CD86 on antigen-presenting cells, and recent studies have shown that abatacept induces reverse signaling in macrophages and osteoclast precursors in a T cell-independent manner. This study aimed to investigate the therapeutic effects of abatacept on circulating monocytes that contribute to RA pathogenesis.

**Methods:**

Purified circulating monocytes derived from RA patients and controls were cultured in the absence or presence of abatacept or CD28-Ig for 24 h. The recovered cells were subjected to flow cytometry to evaluate the expression levels of cell surface molecules, and cytokines and chemokines in the culture supernatant were measured by multiplex bead arrays. The expression of candidate molecules was further examined by immunoblotting using total cellular extracts of the cultured monocytes. Finally, the effects of abatacept on cytokine production in monocytes stimulated with the immune complex of anti-citrullinated peptide antibodies (ACPAs) were examined.

**Results:**

CD64/FcγRI was identified as a monocyte-derived molecule that was downregulated by abatacept but not CD28-Ig. This effect was observed in both RA patients and controls. The abatacept-induced downregulation of CD64/FcγRI was abolished by treatment with anti-CD86 antibodies but not anti-CD80 antibodies. Abatacept suppressed the production of interleukin (IL)-1β, IL-6, C-C motif chemokine ligand 2, and tumor necrosis factor-α in cultured monocytes stimulated with the ACPA immune complex.

**Conclusions:**

The therapeutic effects of abatacept on RA are mediated, in part, by the downregulation of CD64/FcγRI on circulating monocytes via direct binding to CD86 and the suppression of immune complex-mediated inflammatory cytokine production.

**Supplementary Information:**

The online version contains supplementary material available at 10.1186/s13075-022-02886-8.

## Introduction

Rheumatoid arthritis (RA) is an autoimmune disease characterized by chronic polyarthritis that leads to joint destruction, impaired quality of life, and poor outcomes [1]. The production of autoantibodies that react with posttranslationally modified self-antigens, such as anti-citrullinated peptide antibodies (ACPAs), is one of the autoimmune features of RA and often precedes the onset of synovitis [2]. The pathogenic processes in the RA synovium include the infiltration of various immunocompetent cells, the production of inflammatory cytokines, synovial proliferation, neovascularization, and the differentiation and activation of osteoclasts, resulting in osteochondral destruction [3]. During this process, peripheral blood monocytes infiltrate the joint and are involved in the inflammatory pathology mediated by their differentiation into macrophages, bone destruction mediated by their differentiation into osteoclasts, and enhanced adaptive immunity in antigen-presenting cells (APCs) [4]. It has been shown that ACPAs promote synovial inflammation by activating monocytes and macrophages through the formation of an immune complex with citrullinated peptides, binding to Fcγ receptors, and inducing the production of inflammatory cytokines [5].

The management of RA has evolved through the spread of treat-to-target strategies and early interventions with disease-modifying anti-rheumatic drugs (DMARDs). In particular, biologic DMARDs play significant roles in achieving the therapeutic goal of clinical remission, low disease activity, and halting the progression of joint damage in RA patients who previously experienced inadequate responses to conventional synthetic DMARDs [6]. Abatacept, one of the biologic DMARDs, is a fusion protein of the extracellular domain of cytotoxic T-lymphocyte-associated protein 4 (CTLA4) and the Fc portion of human immunoglobulin G (IgG) [7]. Antigen-specific activation of T cells requires costimulation by the interaction between CD28 on T cells and CD80/CD86 on APCs such as dendritic cells and macrophages, in addition to recognition of the antigenic peptides presented on APCs by a T cell receptor [8]. To avoid excessive T cell activation, CTLA4, which is expressed transiently by activated CD4^+^ T cells or constitutively by regulatory T cells, has the ability to suppress the antigen-specific adaptive immune response by competitively inhibiting the interaction between CD28 and CD80/86 [9]. It has been thought that the therapeutic effects of abatacept on RA are mediated by suppressing the upstream autoimmune and inflammatory processes by interfering with CD28 binding to CD80/86 on APCs [10, 11]. On the other hand, it has been recently reported that abatacept exerts a pharmacologic effect directly on monocytes/macrophages and osteoclasts via CD80/86 in a T cell-independent manner [10]. For example, abatacept directly inhibits osteoclastogenesis by suppressing the differentiation of circulating osteoclast precursors into functional osteoclasts, and this process is mediated by receptor activator of NF-κB ligand expressed by osteoblasts or activated T cells [12, 13]. In addition, short-term coculture of synovial macrophages derived from RA patients and Jurkat cells showed that abatacept suppressed in vitro production of inflammatory cytokines, such as interleukin (IL)-6 and tumor necrosis factor (TNF)-α [14]. These findings indicate that abatacept exerts suppressive effects on osteoclast precursors and macrophages by directly binding to CD80/86 on the cell surface. On the other hand, the detailed mechanism by which abatacept acts on peripheral blood monocytes remains unclear. In this study, highly purified monocyte cultures were used to investigate the therapeutic effects of abatacept on circulating monocytes in RA patients.

## Materials and methods

### Patients and controls

Peripheral blood samples were obtained from 57 patients with RA and 12 controls. RA patients were recruited from the outpatient clinics of Nippon Medical School Hospital. The inclusion criteria were fulfillment of the 2010 American College of Rheumatology (ACR)/European League Against Rheumatism (EULAR) classification criteria [15], moderate or high disease activity according to the simplified disease activity index (SDAI) [16], and DMARD-naïve status. Additional criteria, including ACPA positivity and treatment-naive status, were required for inclusion in an assay to measure cytokine production in response to the ACPA-immune complex. The controls included 7 healthy individuals and 5 patients with noninflammatory rheumatic and musculoskeletal diseases, including osteoarthritis and menopausal disorders. Peripheral blood samples from some patients were repeatedly used for experiments. Finally, serum total IgG was purified from 12 patients with RA and high-titer ACPAs (> 500 U/mL) and from 5 healthy individuals who were confirmed to be negative for ACPAs. This study was approved by the Institutional Review Board of Nippon Medical School Hospital (27-10-507 and 30-09-992), and written informed consent was obtained from all subjects.

### Isolation of highly purified peripheral blood monocytes

Peripheral blood mononuclear cells (PBMCs) were isolated from heparinized venous blood using Lymphoprep™ (Axis-Shield PoC AS, Oslo, Norway) density gradient centrifugation. Subsequently, CD14^+^ monocytes were separated from PBMCs using a MACS® negative selection system (Miltenyi Biotech, Bergisch Gladbach, Germany) after the cells were pretreated with an Fc Receptor Blocking Reagent (Miltenyi Biotech) according to the manufacturer’s instructions. To further remove residual T cells, the cells were subsequently subjected to MACS® column separation using anti-CD3 monoclonal antibody (mAb)-coupled magnetic beads (Miltenyi Biotech). The proportion of CD3^−^CD14^+^ cells in the purified monocyte fraction was confirmed by flow cytometry and was consistently > 97%.

### Short-term monocyte cultures

Highly purified CD14^+^ monocytes were resuspended in RPMI 1640 (Sigma–Aldrich, St. Louis, MO, USA) supplemented with 10% heat-inactivated fetal bovine serum and were cultured in 5% CO_2_ for 24 h in the absence (mock) or presence of abatacept (10 μg/mL; Bristol-Myers Squibb, NJ, USA) or recombinant human CD28 Fc chimera (CD28-Ig) (10 μg/mL; R&D systems, MN, USA). Supernatants were recovered, and adherent cells were carefully scraped with phosphate-buffered saline containing 2 mM ethylenediaminetetraacetic acid at 4 °C. For the time course experiment, monocyte cultures with or without abatacept were harvested at 6, 24, or 48 h. In some experiments, abatacept-treated monocytes were cultured in the presence of anti-CD80 mAb/clone 2D10.4, anti-CD86 mAb/clone IT2.2, the combination of anti-CD80 plus anti-CD86 mAbs, or isotype-matched control mAbs (10 μg/mL; Thermo Fisher Scientific, Waltham, MA, USA). In another experiment, recombinant human IgG1-Fc (10 μg/mL; R&D systems) was added to monocyte cultures in the presence of abatacept.

### Monocyte cultures with the ACPA-immune complex

Human fibrinogen (Abcam, Cambridge, UK) was first passed through a protein G column (GE Healthcare, IL, USA) to remove residual IgG and was incubated with 1 mg/mL rabbit peptidylarginine deiminase (PAD; 7 units/mL; Sigma–Aldrich, St. Louis, MO, USA) in reaction buffer (0.1 M Tris-HCl pH 7.4, 10 mM CaCl_2_, and 5 mM dithiothreitol) at 37 °C for 2 h [17]. The solution was replaced with phosphate-buffered saline after ultrafiltration to obtain citrullinated fibrinogen. The IgG fraction was purified from pooled ACPA-positive RA sera and ACPA-negative healthy control sera using a protein G column [18]. PAD-induced citrullination of fibrinogen was assessed by enzyme-linked immunosorbent assay principally as described previously [19]. Briefly, PAD- or mock-treated fibrinogen (100 μg/mL) was coated in duplicate on 96-well polyvinyl plates (Sumilon multi-well plate H type; Sumitomo Bakelite, Tokyo, Japan). After blocking with phosphate-buffered saline containing 3% bovine serum albumin, the wells were incubated with purified ACPA-positive IgG (100 μg/mL), ACPA-negative IgG (100 μg/mL), serum from ACPA-positive RA patient diluted 1:50 or serum from ACPA-negative healthy control diluted 1:50, and subsequently with peroxidase-conjugated anti-human IgG (Jackson ImmunoResearch Laboratories, PA, USA) diluted 1:200,000. After washing, the bound antibodies were visualized by tetramethylbenzidine substrate (Sigma–Aldrich) dissolved in dimethyl sulfoxide and buffered with phosphate-citrate, and the reaction was stopped by the addition of 1N sulfuric acid. The optical density at 450 nm (OD_450_) was then read with a microplate reader (Agilent Technologies, CA, USA).

A 96-well microplate was precoated with citrullinated fibrinogen (20 μg/mL), blocked with phosphate-buffered saline containing 3% bovine serum albumin, and incubated with ACPA-positive or ACPA-negative IgG (100 μg/mL) [19]. Peripheral blood monocytes from ACPA-positive, treatment-naive RA patients were suspended in serum-free macrophage medium (Thermo Fisher Scientific) and seeded on an ACPA immune complex-precoated 96-well microplate at 50,000 cells/well [20]. The cells were treated in the presence or absence of abatacept (10 μg/mL) at 37 °C and 5% CO_2_, and the supernatants were collected after 24 h.

### Multicolor flow cytometry

The surface expression of CD16/FcγRIII, CD32/FcγRII, CD40, CD54, CD62L, CD64/FcγRI, CD80, CD86, CD181/CXCR1, CD182/CXCR2, CD184/CXCR4, CD191/CCR1, CD192/CCR2, CD194/CCR4, CD195/CCR5, CD273/programmed death-ligand (PD-L) 2, CD274/PD-L1, CD275/inducible T cell costimulator ligand, CX3CR1, and human leukocyte antigen (HLA)-DR on cultured monocytes was analyzed on a FACSCalibur™ flow cytometer using the CellQuest™ Pro software (BD Biosciences, Franklin Lakes, NJ, USA). These molecules were selected on the basis of their potential involvement in the pathogenic process of RA, as reported previously (Additional file 1: Table S1). Viable cells were identified by gating on forward and side scatters, and the data are shown as logarithmic dot plots or histograms. The expression levels of individual cell surface molecules on the gated CD14^+^ cells were analyzed and are shown as the mean fluorescence intensity (MFI) ratio, which was calculated by dividing the MFI of the cells stained with mAbs of interest by the MFI of the cells stained with corresponding isotype-matched control mAbs.

### Measurement of cytokines and chemokines

The concentrations of IL-1β, IL-6, IL-8, IL-10, IL-12p70, interferon (IFN)-γ, C-C motif chemokine ligand 2 (CCL2), and TNF-α in the culture supernatant were measured by a BD™ Cytometric Bead Array (Human CBA FLEX Sets, BD Biosciences) according to the manufacturer’s instructions. Briefly, culture supernatants were incubated with the cytokine bead mixture and were analyzed on a FACSCalibur™ flow cytometer using quantification software (FCAP Array™ v3.0; BD Biosciences).

### Immunoblotting

Cultured monocytes were subjected to 10% polyacrylamide-sodium dodecyl sulfate gel electrophoresis, followed by blotting onto nitrocellulose membranes. The membranes were incubated first with 3% bovine serum albumin in 0.05% Tween 20 in Tris-buffered saline and subsequently with rabbit anti-CD16 polyclonal antibodies, anti-CD32a/FcγRIIa polyclonal antibodies, anti-CD32b/FcγRIIb mAb/clone EP888Y, anti-CD64/FcγRI mAb/clone EPR4623, anti-CD80 mAb/clone EPR1157, or anti-CD86 mAb/clone EP1158Y (all purchased from Abcam). Mouse anti-β-actin mAb/clone AC-74 (Sigma–Aldrich) was used as a loading control. After being incubated with horseradish peroxidase-conjugated anti-rabbit or anti-mouse IgG (Thermo Fisher) secondary antibodies, antibody binding was visualized using an enhanced chemiluminescence kit (Western Lighting Plus-ECL; PerkinElmer, Waltham, MA). The molecules of interest were identified on the basis of the reported molecular sizes. The signal intensity of each protein band was quantified using the ImageJ software (https://imagej.nih.gov/ij/) and is shown as the intensity ratio, which was calculated by dividing the intensity of the corresponding bands by the intensity of the β-actin band.

### Statistical analysis

All continuous values are shown as the mean ± standard deviation (SD) or median and interquartile range. Comparisons between the two groups were analyzed for statistical significance using the nonparametric Mann–Whitney *U* test with IBM SPSS Statistics version 23.0 (IBM, New York, NY, USA).

## Results

### Screening of monocyte-derived molecules that exhibit differential expression specifically after exposure to abatacept

The demographic and clinical features of DMARD-naïve RA patients and controls used for the derivation or validation experiments to screen for monocyte-derived molecules that were differentially expressed after exposure to abatacept are listed in Additional file 1: Table S2. The majority of RA patients had early disease with a median disease duration of approximately 2 months and minimal joint destruction (Steinbrocker stage I ≥ 70%). Age and gender were not matched between RA patients and controls in the screening, while RA patients and controls were more balanced for age and gender in the validation experiments.

To screen the molecules that showed changes in their expression after short-term exposure to abatacept, peripheral blood monocytes from 5 RA patients and 5 controls were used in a derivation experiment, which involved 20 cell surface molecules (Additional file 1: Fig. S1) and 8 cytokines/chemokines (Additional file 1: Fig. S2). IL-12p70 was detectable in none of the samples. As a result, CD64/FcγRI, CD80, CD86, and CXCR2 were selected as candidate molecules based on statistically significant downregulation or upregulation in RA patient or control monocyte cultures with abatacept compared with the mock cultures (*P* < 0.1).

**Fig. 1:**
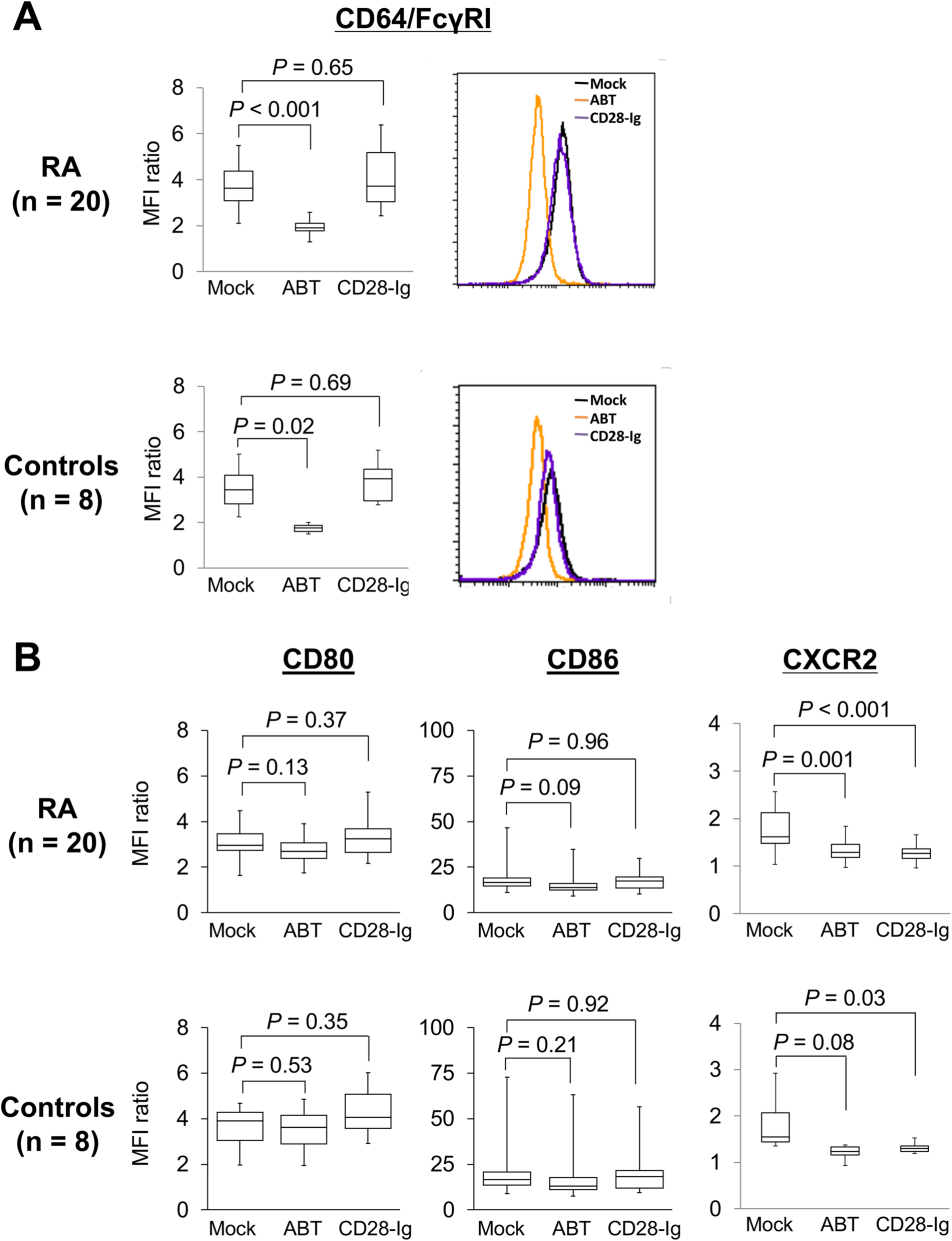
Downregulation of CD64/FcγRI on monocytes after exposure to abatacept, as shown by flow cytometry. Peripheral blood monocytes were cultured for 24 h in the absence (mock) or presence of abatacept (ABT) or CD28-Ig and the expression of CD64/FcγRI (**A**) and CD80, CD86, and CXCR2 (**B**) was examined by flow cytometry. Peripheral blood monocytes were obtained from 20 patients with RA and 8 controls and used for a validation experiment. The cell surface expression is expressed as the mean fluorescence intensity (MFI) ratio. A representative histogram showing the expression of CD64/FcγRI in a patient with RA and a control is also shown. The results are shown in boxplots, and statistical comparisons between the two groups were made using the Mann–Whitney *U* test

Changes in the expression of CD64/FcγRI, CD80, CD86, and CXCR2 were further analyzed in a validation experiment using peripheral blood monocytes from 20 patients with RA and 8 controls (Fig. 1). The expression levels of CD64/FcγRI in cultures with abatacept were significantly lower than those of mock cultures of cells from RA patients (*P* < 0.001) and controls (*P* = 0.02). In contrast, there was no difference in the expression levels between cultures containing CD28-Ig and mock cultures, indicating that the change in CD64/FcγRI expression was specific to abatacept exposure. The expression levels of CD64/FcγRI in mock cultures were similar between RA patients and the controls (3.95 ± 1.89 versus 3.50 ± 0.97), and abatacept-induced downregulation of CD64/FcγRI was observed regardless of the presence or absence of RA. A time course experiment using peripheral blood monocytes from 3 controls revealed that the downregulation of CD64/FcγRI expression could be observed at 6 h in the presence of abatacept and was sustained for at least 48 h (Additional file 1: Fig. S3). On the other hand, there was no statistically significant difference in the expression levels of CD80 and CD86 in cultures with abatacept compared with mock cultures, while there was a trend toward downregulation of CD80 and CD86 after abatacept exposure in RA monocytes (*P* = 0.13 and 0.09, respectively). Since quantitative evaluation of CD80 and CD86 by flow cytometry may not be accurate because abatacept may interfere with the binding of anti-CD80 or anti-CD86 mAbs, we decided to further examine the expression of CD80 and CD86 by immunoblot analysis of whole cell lysates. Finally, the expression levels of CXCR2 were decreased in cultures with abatacept compared with mock cultures (*P* = 0.001 and 0.08 for RA and controls, respectively), whereas the expression of CXCR2 was also decreased in cultures with CD28-Ig compared with mock cultures (*P* < 0.001 and *P* = 0.03 for RA and controls, respectively), indicating that the downregulation of CXCR2 was not specific to exposure to abatacept.

Abatacept may interfere with the detection of CD64/FcγRI by flow cytometry due to its binding to CD64/FcγRI via the IgG1-Fc portion, although this was unlikely because the IgG1-Fc portion of abatacept was modified by amino acid substitution to reduce its binding affinity for Fcγ receptors [21], and Fc Receptor Blocking Reagent was used during the monocyte isolation procedure. To eliminate this possibility, the expression levels of CD64/FcγRI in cultures of monocytes derived from 5 patients with RA in the absence or presence of abatacept or intact human IgG1-Fc were examined by flow cytometry. CD64/FcγRI expression was not influenced by the presence of human IgG1-Fc but was decreased by the presence of abatacept (Additional file 1: Fig. S4).

### Confirmation of abatacept-induced downregulation of CD64/FcγRI on monocytes by immunoblots

**Fig. 2:**
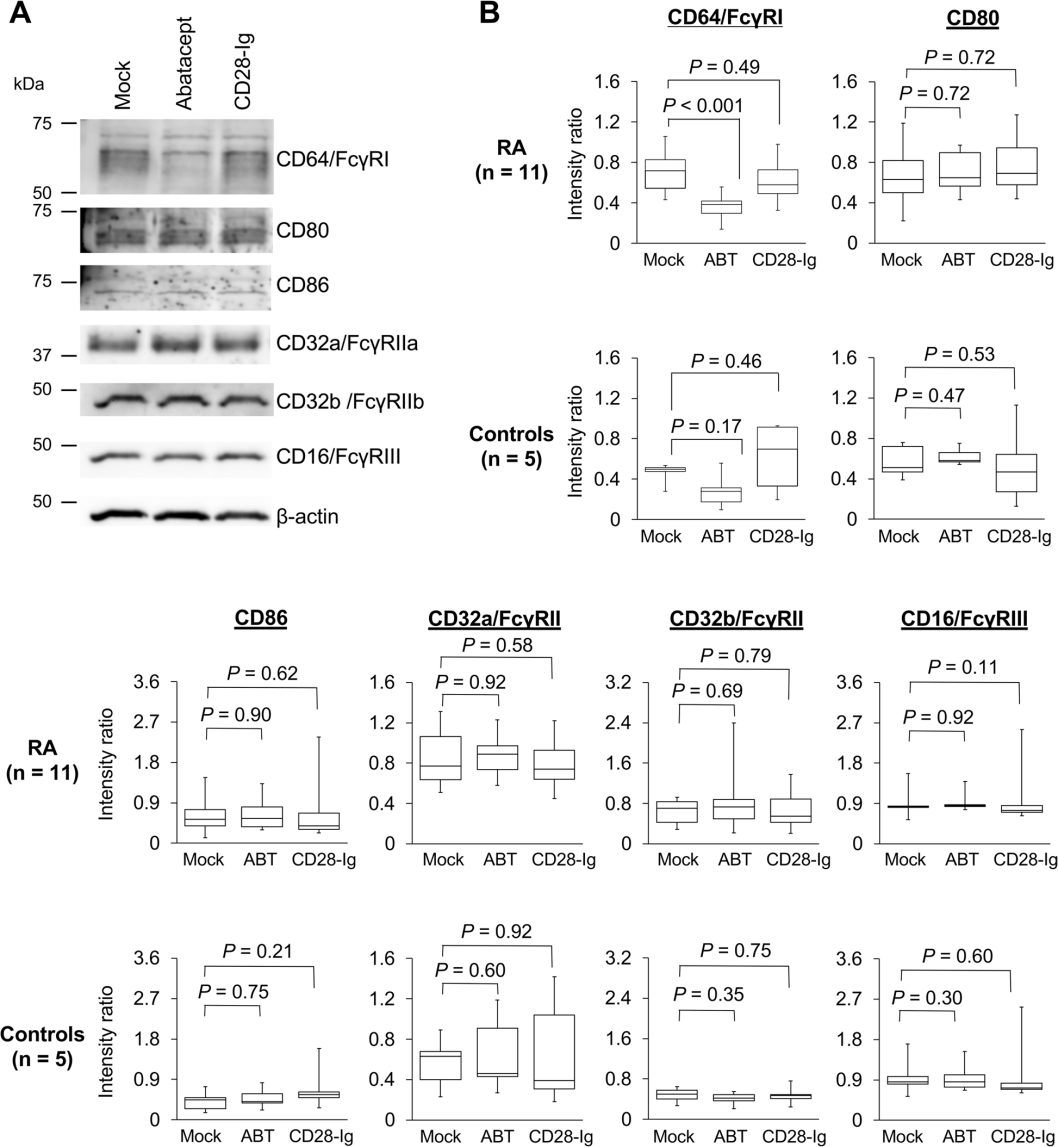
Downregulation of CD64/FcγRI on monocytes after exposure to abatacept, as shown by immunoblotting. Peripheral blood samples were obtained from 11 patients with RA and 5 controls and used for immunoblotting. The protein expression of CD64/FcγRI, CD80, CD86, CD32a/FcγRIIa, CD32b/FcγRIIb, and CD16/FcγRIII in whole-cell extracts of monocytes cultured for 24 h in the absence (mock) or presence of abatacept or CD28-Ig. A representative immunoblot image showing a patient with RA in the right panel with the positions of molecular weight markers (**A**). The signal intensity of individual protein bands was quantified using the ImageJ software and is shown as the intensity ratio, which was calculated by dividing the intensity of the band of interest by the intensity of the β-actin band (**B**). The results are shown in boxplots, and the statistical comparisons between the two groups were made using the Mann–Whitney *U* test

The protein expression of CD64/FcγRI was further examined by immunoblot analysis of whole-cell lysates of monocytes cultured in the absence (mock) or presence of abatacept or CD28-Ig. The expression of additional Fcγ receptors (CD16/FcγRIII, CD32a/FcγRIIa, and CD32b/FcγRIIb) and ligands for CTLA4 (CD80 and CD86) was also examined (Fig. 2). The downregulation of CD64/FcγRI induced by abatacept but not CD28-Ig was reproduced in peripheral blood monocytes derived from 11 RA patients (*P* < 0.001). Immunoblot analysis of peripheral blood monocytes from 5 controls showed the same trend, but the difference was not statistically significant (*P* = 0.17). In mock cultures, there was a trend toward higher expression of CD64/FcγRI in RA patients than in controls (0.70 ± 0.21 versus 0.46 ± 0.10, *P* = 0.07). In contrast, there was no difference in the protein expression levels of CD80, CD86, CD32a/FcγRIIa, CD32b/FcγRIIb, or CD16/FcγRIII between mock cultures and cultures with abatacept or CD28-Ig.

### Downregulation of CD64/FcγRI via abatacept binding to CD86 on monocytes

**Fig. 3:**
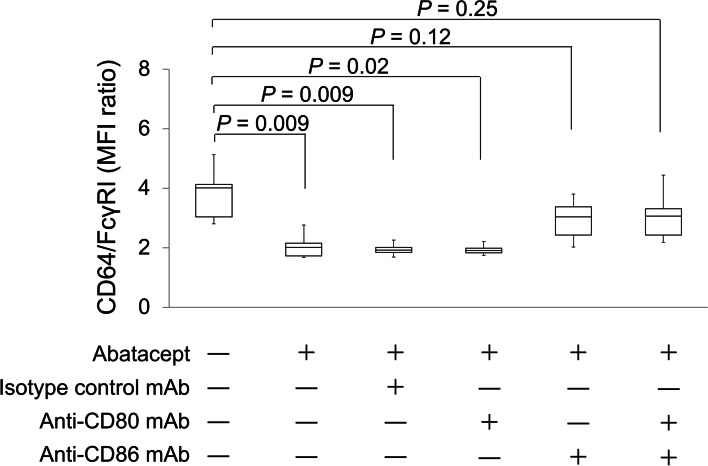
Downregulation of CD64/FcγRI via abatacept binding to CD86 on monocytes. The effects of anti-CD80, anti-CD86, both mAbs, or isotype-matched mAbs on abatacept-induced downregulation of CD64/FcγRI were examined in a short-term monocyte culture. Peripheral blood samples were obtained from 5 patients with RA and were used. The cell surface expression of CD64/FcγR is expressed as the mean fluorescence intensity (MFI) ratio. The results are shown in boxplots, and statistical comparisons between the two groups were made using the Mann–Whitney *U* test. A representative result of 3 independent experiments is shown

To investigate which CTLA4 ligand (CD80 or CD86) was involved in the abatacept-induced downregulation of CD64/FcγRI on monocytes, peripheral blood monocytes from 5 patients with RA were cultured with abatacept in the presence or absence of anti-CD80 mAb, anti-CD86 mAb, a combination of anti-CD80 plus anti-CD86 mAbs, or isotype-control mAb (Fig. 3). The abatacept-induced downregulation of CD64/FcγRI was abolished in the presence of anti-CD86 mAb but not anti-CD80 mAb alone, indicating that CD86 was responsible for binding to abatacept and the resultant downregulation of CD64/FcγRI on monocytes.

### Effects of abatacept on cytokine/chemokine production from monocytes stimulated with the ACPA immune complex

**Fig. 4:**
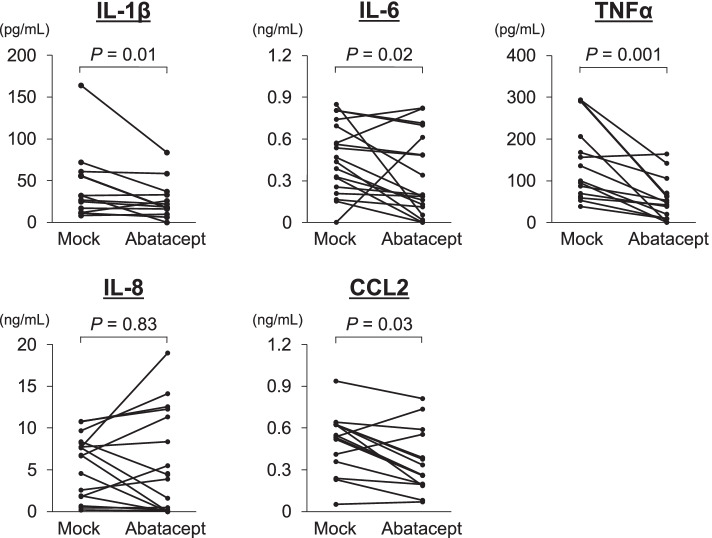
Effects of abatacept on cytokine/chemokine production in monocytes stimulated with the ACPA immune complex. Peripheral blood samples were obtained from 17 patients with rheumatoid arthritis and used. The concentrations of IL-1β, IL-6, TNF-α, IL-8, and CCL2 were measured by multiplex bead arrays. Statistical comparisons between the two groups were made using the Mann–Whitney *U* test

Since it has been reported that macrophages derived from the peripheral blood monocytes of RA patients produce inflammatory cytokines and chemokines after binding to the ACPA immune complex [22], abatacept-induced downregulation of CD64/FcγRI on monocytes may suppress ACPA immune complex-induced production of inflammatory cytokines/chemokines in patients with ACPA-positive RA. To test this hypothesis, peripheral blood monocytes were isolated from 19 RA patients who were ACPA-positive and treatment-naive and were cultured with ACPA immune complexes in the absence (mock) or presence of abatacept. PAD-induced citrullination of fibrinogen used for this assay was confirmed by specific recognition of citrullinated fibrinogen by ACPA-positive IgG in enzyme-linked immunosorbent assay (Additional file 1: Fig. S5). Two patients were excluded because none of the cytokines/chemokines was detectable in the supernatants of the mock cultures. IL-10, IL-12p70, and IFN-γ were not detected in the remaining culture supernatants derived from 17 patients. The levels of IL-1β, IL-6, CCL2, and TNF-α were significantly lower in the cultures that were treated with abatacept than in mock cultures (*P* = 0.01, 0.02, 0.001, and 0.03, respectively), whereas there was no difference in the level of IL-8 among the cultures (Fig. 4).

## Discussion

We have demonstrated that abatacept binds to CD86 on peripheral blood monocytes and downregulates the expression of CD64/FcγRI within 24 h in a T cell-independent manner. In addition, abatacept is capable of suppressing the ACPA immune complex-induced production of inflammatory cytokines/chemokines such as TNF-α and IL-6 in monocytes. These T cell-independent mechanisms of abatacept may contribute to the suppression of RA pathogenesis, although there is no direct evidence linking the downregulation of CD64/FcγRI to the suppression of cytokine/chemokine production. This rapid effect might be one explanation for the relatively rapid improvement in disease activity indices after the administration of abatacept, which are comparable to the effects of TNF inhibitors [23]. The ability of abatacept to suppress the ACPA immune complex-induced production of inflammatory cytokines/chemokines from monocytes may also explain why the clinical efficacy of abatacept was more prominent in ACPA-positive RA patients than in ACPA-negative patients, and RA patients with higher ACPA titers showed better responses to abatacept [24].

It has been reported that circulating monocytes from RA patients exhibit higher levels of activating FcγRs, including CD64/FcγRI, than those from healthy controls [25]. This trend was observed in our study by immunoblot analysis using whole-monocyte extracts. Decreased expression of CD64/FcγRI on peripheral blood monocytes has been reported in RA patients who were treated with methotrexate or TNF inhibitors, especially those who were good responders [26, 27], suggesting a relationship between CD64/FcγRI expression on monocytes and RA disease activity. However, anti-TNF-α mAbs failed to downregulate CD64/FcγRI expression on monocytes in vitro, suggesting that the decrease in CD64/FcγRI expression on monocytes was an indirect effect of TNF-α blockade on disease activity [27]. The capacity to directly downregulate CD64/FcγRI expression on peripheral blood monocytes might be a unique effect of abatacept.

In our study, abatacept suppressed the production of inflammatory cytokines in monocyte cultures stimulated with the ACPA immune complex. This suppressive effect of abatacept was not observed in cultures of monocytes alone, suggesting that the effect of abatacept may be mediated through the downregulation of CD64/FcγRI expression and decreased uptake of the ACPA immune complex by monocytes. This effect is analogous to a previous report showing that the ACPA immune complex-induced production of IL-1β, IL-6, IL-8, CCL2, and TNF-α was suppressed by treatment with abatacept in cultures of macrophages that were generated in vitro from CD68^+^ peripheral blood monocytes from RA patients in the presence of macrophage colony-stimulating factor [28]. However, a recent study using macrophages that originated from the peripheral blood monocytes of RA patients showed that low-affinity CD32a/FcγRIIa was the main Fcγ receptor for capturing the ACPA immune complex and promoting TNF-α production [20]. The difference between this finding and our results may be explained by substantial differences in the expression profiles of Fcγ receptors on monocytes and macrophages: the expression of CD64/FcγRI was higher on monocytes than on macrophages [29].

CD64/FcγRI expressed on circulating monocytes is also involved in the differentiation of circulating monocytes into functional dendritic cells. Specifically, phagocytosis of the IgG immune complex via CD64/FcγRI enhances the cell surface expression of HLA class II molecules and differentiates monocytes into dendritic cells with increased abilities to induce the priming and expansion of antigen-specific T cells [30, 31]. Overall, the downregulation of CD64/FcγRI expression on peripheral blood monocytes induced by abatacept may contribute to the suppression of RA pathology through multiple mechanisms.

This study focused on CD64/FcγRI as a cell-surface molecule that downregulated after exposure to abatacept. Other monocyte-derived molecules, such as CD15, CD54, CD80, and CD102, were shown to be downregulated upon abatacept exposure in in vitro monocyte cultures, but these findings were not necessarily conformed by others [10, 32–34]. In this regard, a trend toward abatacept-induced suppression of cell-surface expression of CD54 and CD80 was observed in our study, which may not be sensitive enough to detect small changes in the screening experiments due to a small number of subjects analyzed. It is likely that differences in experimental conditions, i.e., sources of monocytes (disease duration, disease activity, and use of DMARDs in RA patients), monocyte culture conditions, and methods for qualifying expression levels (flow cytometry, immunoblots, and quantitative PCR), influence the results. More importantly, it has been shown that a significant reduction in the expression of several adhesion molecules of monocytes induced by abatacept led to a reduced adhesion of monocytes to endothelial cells, contributing to a diminished inflammation in the synovial tissue of the joints [33]. Cutolo et al. recently reported that abatacept treatment induced a shift from M1 to M2 macrophages in vitro in cultures of monocyte-derived macrophages derived from RA patients and healthy donors [35]. Since the differential screening of M1-specific and M2-specific surface phenotypes using human PBMC-derived macrophages identified CD64/FcγRI as a molecule that was upregulated on M1 compared with M2 macrophages [36], the downregulation of CD64/FcγRI observed in our study can be explained by a shift from the M1 to the M2 phenotype.

There are limitations in this study. Current data did not prove direct evidence showing that the effects of abatacept on cytokine/chemokine production from monocytes are mediated through downregulation of CD64/FcγRI. Experiments using of Toll-like receptor ligands and non-ACPA immune complex as potential monocyte stimulants, instead of the ACPA immune complex, in in vitro cultures may provide useful insights into the mechanisms underlying the abatacept-induced suppression of ACPA immune complex-mediated inflammatory cytokine production. In addition, our findings were obtained in in vitro short-term cultures of peripheral blood monocytes, and it is not clear if the proposed mechanism is truly exerted in RA patients. Further studies to investigate chronological changes in peripheral blood monocytic phenotypes and cytokine production in ACPA-positive and ACPA-negative RA patients treated with or without abatacept are necessary to confirm the in vivo therapeutic effects of abatacept.

## Conclusions

The results of this in vitro analysis suggest that abatacept directly downregulates CD64/FcγRI expression on peripheral blood monocytes by reverse signaling through CD86 and suppresses ACPA immune complex-mediated inflammatory cytokine production. Our findings are useful for understanding the unique therapeutic mode of action of abatacept, which has multifaceted effects unlike other biologic DMARDs and is expected to contribute to the adequate use of abatacept in clinical practice.

## Supplementary Information

**Additional file 1: Table S1.** A list of cell surface molecules investigated and monoclonal antibodies used for flow cytometry. **Table S2.** Demographic and clinical features of RA patients and controls used in the series of experiments. **Figure S1.** Screening of monocyte-derived cell surface molecules that were differentially expressed after short-term culture with abatacept. Peripheral blood samples from 5 patients with rheumatoid arthritis (RA) and 5 controls were used for a derivation experiment. Circulating monocytes were cultured for 24 hours in the absence (mock) or presence of abatacept (ABT) or CD28-Ig. Cell surface expression was quantified by flow cytometry and is expressed as the mean fluorescence intensity (MFI) ratio. Statistical comparisons between two groups were made using the Mann–Whitney *U* test. **Figure S2.** Screening of monocyte-derived cytokines/chemokines that were differentially expressed after short-term culture with abatacept. Peripheral blood samples from 5 patients with rheumatoid arthritis (RA) and 5 controls were used for a derivation experiment. Circulating monocytes were cultured for 24 hours in the absence (mock) or presence of abatacept (ABT) or CD28-Ig. The concentrations of interleukin (IL)-1β, IL-6, IL-8, IL-10, C-C motif chemokine ligand 2 (CCL2), and tumor necrosis factor (TNF)-α were measured by multiplex bead arrays. Statistical comparisons between two groups were made using the Mann–Whitney *U* test. **Figure S3.** Time course of abatacept-induced downregulation of CD64/FcγRI on monocytes. Peripheral blood samples were obtained from 3 controls and cultured with or without abatacept for 6, 24, and 28 hours. The expression levels of CD64/FcγRI were quantified by flow cytometry and are expressed as the CD64/FcγRI expression ratio, which was calculated by dividing the mean fluorescence intensity ratio of monocytes cultured with abatacept by the mean fluorescence intensity ratio of monocytes cultured without abatacept. The results are shown as the mean and standard deviation. A representative result of 2 independent experiments is shown. **Figure S4.** Abatacept-induced downregulation of CD64/FcγRI on monocytes is independent of the IgG1-Fc portion of abatacept. Peripheral blood monocytes were obtained from 5 patients with rheumatoid arthritis (RA) and cultured without (mock) or with abatacept or intact human IgG1-Fc for 24 hours. The expression levels of CD64/FcγRI were quantified by flow cytometry and are expressed as the mean fluorescence intensity (MFI) ratio. The results are shown in boxplots, and statistical comparisons between two groups were made using the Mann–Whitney *U* test. A representative result of 3 independent experiments is shown. **Figure S5.** Confirmation of the peptidylarginine deiminase (PAD)-induced citrullination of fibrinogen by specific recognition of ACPA-positive IgG in enzyme-linked immunosorbent assay. PAD- or mock-treated fibrinogen was coated in duplicate on 96-well polyvinyl plates, and applied for enzyme-linked immunosorbent assay using purified ACPA-positive IgG, ACPA-negative IgG, serum from ACPA-positive RA patient or serum from ACPA-negative healthy control. All measurement was conducted in duplicate, and results are shown as mean ± standard deviation. A representative result of 3 independent experiments is shown.

## Data Availability

The datasets analyzed during the current study are available from the corresponding author upon reasonable request.

## Abbreviations

RA: Rheumatoid arthritis
ACPA: Anti-citrullinated peptide antibody
APC: Antigen-presenting cell
DMARDs: Disease-modifying anti-rheumatic drugs
CTLA4: Cytotoxic T-lymphocyte-associated protein 4
IL: Interleukin
TNF: Tumor necrosis factor
mAb: Monoclonal antibody
Ig: Immunoglobulin
HLA: Human leukocyte antigen
MFI: Mean fluorescence intensity
IFN: Interferon
PD-L: Programmed death ligand
PAD: Peptidylarginine deiminase
OD_450_: Optical density at 450 nm
