# Supplementary material for: Abatacept downregulates Fcγ receptor I on circulating monocytes: a potential therapeutic mechanism in patients with rheumatoid arthritis
Source: Arthritis Res Ther. 2022 Aug 13;24:194. doi: 10.1186/s13075-022-02886-8 (PMC9375333; doi:10.1186/s13075-022-02886-8)
Supplement: Supplementary file 1 — Additional file 1: Table S1. A list of cell surface molecules investigated and monoclonal antibodies used for flow cytometry. Table S2. Demographic and clinical features of RA patients and controls used in the series of experiments. Figure S1. Screening of monocyte-derived cell surface molecules that were differentially expressed after short-term culture with abatacept. Peripheral blood samples from 5 patients with rheumatoid arthritis (RA) and 5 controls were used for a derivation experiment. Circulating monocytes were cultured for 24 hours in the absence (mock) or presence of abatacept (ABT) or CD28-Ig. Cell surface expression was quantified by flow cytometry and is expressed as the mean fluorescence intensity (MFI) ratio. Statistical comparisons between two groups were made using the Mann–Whitney U test. Figure S2. Screening of monocyte-derived cytokines/chemokines that were differentially expressed after short-term culture with abatacept. Peripheral blood samples from 5 patients with rheumatoid arthritis (RA) and 5 controls were used for a derivation experiment. Circulating monocytes were cultured for 24 hours in the absence (mock) or presence of abatacept (ABT) or CD28-Ig. The concentrations of interleukin (IL)-1β, IL-6, IL-8, IL-10, C-C motif chemokine ligand 2 (CCL2), and tumor necrosis factor (TNF)-α were measured by multiplex bead arrays. Statistical comparisons between two groups were made using the Mann–Whitney U test. Figure S3. Time course of abatacept-induced downregulation of CD64/FcγRI on monocytes. Peripheral blood samples were obtained from 3 controls and cultured with or without abatacept for 6, 24, and 28 hours. The expression levels of CD64/FcγRI were quantified by flow cytometry and are expressed as the CD64/FcγRI expression ratio, which was calculated by dividing the mean fluorescence intensity ratio of monocytes cultured with abatacept by the mean fluorescence intensity ratio of monocytes cultured without abatacept. The results are shown as t [file 13075_2022_2886_MOESM1_ESM.docx]

**Table S1.** A list of cell surface molecules investigated and monoclonal antibodies used for flow cytometry

| Cell surface molecules | mAb clone | Isotope | Fluorescent label | Manufacturer |
| --- | --- | --- | --- | --- |
| CD16/FcγRIII^1)^ | 3G8 | mouse IgG1 | FITC | BD Biosciences |
| CD32/FcγRII^1)^ | FLI8.26 | mouse IgG2b | FITC | BD Biosciences |
| CD40^2)^ | MAB89 | mouse IgG1 | PE | Beckman Coulter |
| CD54^2)^ | 84H10 | mouse IgG1 | PE | Beckman Coulter |
| CD62L^3)^ | DREG56 | mouse IgG1 | FITC | Beckman Coulter |
| CD64/FcγRI^1)^ | 10.1 | mouse IgG1 | FITC | BD Biosciences |
| CD80^2)^ | MAB104 | mouse IgG1 | FITC | Beckman Coulter |
| CD86^2)^ | FM95 | mouse IgG1 | PE | Miltenyi Biotec |
| CD181/CXCR1^4)^ | 29E.2A3 | mouse IgG2b | PE/Cy7 | BioLegend |
| CD182/CXCR2^4)^ | 48311 | mouse IgG2a | PE | R&D systems |
| CD184/CXCR4^4)^ | 12G5 | mouse IgG2a | PE | Beckman Coulter |
| CD191/CCR1^4)^ | 53504 | mouse IgG2b | Alexa Fluor 647 | BD Biosciences |
| CD192/CCR2^1)^ | 48607 | mouse IgG2b | Alexa Fluor 647 | BD Biosciences |
| CD194/CCR4^1)^ | 205410 | mouse IgG2b | FITC | R&D systems |
| CD195/CCR5^1)^ | 2D7/CCR5 | mouse IgG2a | FITC | BD Biosciences |
| CD273/PD-L2^5)^ | MIH18 | mouse IgG1 | APC | BioLegend |
| CD274/PD-L1^5)^ | 29E.2A3 | mouse IgG2b | PE/Cy7 | BioLegend |
| CD275/ICOSL^1)^ | MIH12 | mouse IgG1 | APC | Miltenyi Biotec |
| CX3CR1^6)^ | 2A9-1 | rat IgG2b | PE | BML |
| HLA-DR^6)^ | AC122 | mouse IgG2a | PE | Miltenyi Biotec |

FITC: fluorescein isothiocyanate; PE: phycoerythrin; APC: allophycocyanin; Cy7: cyanine 7; PD-L: programmed death ligand; ICOSL: inducible T-cell costimulator ligand; HLA: human leukocyte antigen.

**References**

1) Roberts CA, Dickinson AK, Taams LS. Front Immunol. 2015; 6:571

2) Bonelli M, Ferner E, Göschl L et al. Arthritis Rheum. 2013; 65: 599-607

3) Khanniche A, Zhou L, Jiang B et al. Front Immunol. 2019; 10: 887

4) Schmutz C, Hulme A, Burman A et al. Arthritis Res Ther. 2005; 7: R217-29

5) Greisen SR Kragstrup TW, Thomsen JS et al. J Transl Autoimmun. 2019; 3: 100028

6) Rana AK, Li Y, Dang Q, Yang F. Int Immunopharmacol. 2018; 65: 348-59

**Table S2.** Demographic and clinical features of RA patients and controls used in the series of experiments

| Demographic and clinical features | Experiments assessing changes in the expression of monocyte-derived molecules after abatacept exposure | | | | Experiments of cytokine production in response to an the ACPA-immune complex |
| --- | --- | --- | --- | --- | --- |
|  | Derivation experiment | | Validation experiment | |  |
|  | RA  (n = 5) | Controls  (n = 5) | RA  (n = 20) | Controls  (n = 8) | RA  (n = 19) |
| Age, years | 66.0 ± 14.4 | 36.2 ± 2.7 | 64.6 ± 16.7 | 52.0 ± 10.3 | 66.7 ± 16.9 |
| Female, n (%) | 5 (100) | 1 (20) | 15 (75) | 6 (75) | 14 (70) |
| Duration of RA, months | 2 (1-3) | N/A | 5.5 (2.3-17.5) | N/A | 2.6 ± 1.6 |
| Smoking history, n (%) | 1 (20) | 0 (0) | 11 (55) | 1 (13) | 9 (47) |
| Steinbrocker stage, n (%) | | | | | |
| Stage I | 4 (80) | N/A | 14 (70) | N/A | 16 (84) |
| Stage II | 0 | N/A | 4 (20) | N/A | 3 (16) |
| Stage III | 0 | N/A | 1 (5) | N/A | 0 |
| Stage IV | 1 (20) | N/A | 1 (5) | N/A | 0 |
| Interstitial lung disease, n (%) | 5 (100) | N/A | 8 (40) | N/A | 4 (21) |
| ACPA titer, IU/mL | 491 (301-662) | N/A | 181 (1.2, 1027) | N/A | 167 (30, 1257) |
| RF titer, U/mL | 327 ± 572 | N/A | 95 ± 142 | N/A | 89 ± 62 |
| MMP-3, ng/mL | 230 ± 123 | N/A | 170 ± 136 | N/A | 243 ± 300 |
| CRP, mg/dL | 6.1 ± 7.2 | N/A | 1.6 ± 2.5 | N/A | 3.5 ± 3.9 |
| SDAI | 25.2 ± 8.8 | N/A | 15.4 ± 10.3 | N/A | 27.8 ± 14.6 |

Continuous values are shown as the mean ± standard deviation or median and interquartile range.

RA: rheumatoid arthritis; N/A: not applicable; ACPA: anti-citrullinated peptide antibody; RF: rheumatoid factor; MMP-3: matrix metalloproteinase 3; SDAI: simplified disease activity index.

**Figure S1.** Screening of monocyte-derived cell surface molecules that were differentially expressed after short-term culture with abatacept. Peripheral blood samples from 5 patients with rheumatoid arthritis (RA) and 5 controls were used for a derivation experiment. Circulating monocytes were cultured for 24 hours in the absence (mock) or presence of abatacept (ABT) or CD28-Ig. Cell surface expression was quantified by flow cytometry and is expressed as the mean fluorescence intensity (MFI) ratio. Statistical comparisons between two groups were made using the Mann–Whitney *U* test.

**Figure S2.** Screening of monocyte-derived cytokines/chemokines that were differentially expressed after short-term culture with abatacept. Peripheral blood samples from 5 patients with rheumatoid arthritis (RA) and 5 controls were used for a derivation experiment. Circulating monocytes were cultured for 24 hours in the absence (mock) or presence of abatacept (ABT) or CD28-Ig. The concentrations of interleukin (IL)-1β, IL-6, IL-8, IL-10, IL-12p70, C-C motif chemokine ligand 2 (CCL2), and tumor necrosis factor (TNF)-α were measured by multiplex bead arrays. IL-12p70 was detectable in none of the samples, and is not shown. Statistical comparisons between two groups were made using the Mann–Whitney *U* test.

**Figure S3.** Time course of abatacept-induced downregulation of CD64/FcγRI on monocytes. Peripheral blood samples were obtained from 3 controls and cultured with or without abatacept for 6, 24, and 28 hours. The expression levels of CD64/FcγRI were quantified by flow cytometry and are expressed as the CD64/FcγRI expression ratio, which was calculated by dividing the mean fluorescence intensity ratio of monocytes cultured with abatacept by the mean fluorescence intensity ratio of monocytes cultured without abatacept. The results are shown as the mean and standard deviation. A representative result of 2 independent experiments is shown.

**Figure S4.** Abatacept-induced downregulation of CD64/FcγRI on monocytes is independent of the IgG1-Fc portion of abatacept. Peripheral blood monocytes were obtained from 5 patients with rheumatoid arthritis (RA) and cultured without (mock) or with abatacept or intact human IgG1-Fc for 24 hours. The expression levels of CD64/FcγRI were quantified by flow cytometry and are expressed as the mean fluorescence intensity (MFI) ratio. The results are shown in boxplots, and statistical comparisons between two groups were made using the Mann–Whitney *U* test. A representative result of 3 independent experiments is shown.

**Figure S5.** Confirmation of the peptidylarginine deiminase (PAD)-induced citrullination of fibrinogen by specific recognition of ACPA-positive IgG in enzyme-linked immunosorbent assay. PAD- or mock-treated fibrinogen was coated in duplicate on 96-well polyvinyl plates, and applied for enzyme-linked immunosorbent assay using purified ACPA-positive IgG, ACPA-negative IgG, serum from ACPA-positive RA patient or serum from ACPA-negative healthy control. All measurement was conducted in duplicate, and results are shown as mean ± standard deviation. A representative result of 3 independent experiments is shown.
